# Inhibitory Effect and Potential Antagonistic Mechanism of Isolated Epiphytic Yeasts against *Botrytis cinerea* and *Alternaria alternata* in Postharvest Blueberry Fruits

**DOI:** 10.3390/foods13091334

**Published:** 2024-04-26

**Authors:** Jia Li, Ting Yang, Furong Yuan, Xinyue Lv, Yahan Zhou

**Affiliations:** School of Light Industry Science and Engineering, Beijing Technology and Business University, Beijing 100048, China; 18401784099@163.com (J.L.); yt18696563546@163.com (T.Y.); yuanfurong0827@126.com (F.Y.); lvxy010605@126.com (X.L.)

**Keywords:** blueberry fruit, epiphytic yeasts, postharvest diseases, lytic enzymes, VOCs, biofilm formation

## Abstract

This study evaluated the biocontrol effect of isolated epiphytic yeasts (*Papiliotrema terrestris, Hanseniaspora uvarum*, and *Rhodosporidium glutinis*) against *Botrytis cinerea* and *Alternaria alternata* in blueberry fruits and its possible mechanisms. Our findings indicated that the three tested yeasts exerted a good biocontrol effect on postharvest diseases in blueberry, and that *H. uvarum* was the most effective. In addition, the three tested yeasts could improve the postharvest storage quality of blueberry fruits to some extent. *H. uvarum* demonstrated the strongest direct inhibitory effect on pathogens by suppressing spore germination, mycelial growth, and antifungal volatile organic compound (VOC) production. *P. terrestris* showed the highest extracellular lytic enzymes activities. It also had better adaptation to low temperature in fruit wounds at 4 °C. The biofilm formation capacity was suggested to be the main action mechanism of *R. glutinis*, which rapidly colonized fruit wounds at 20 °C. Several action mechanisms are employed by the superb biocontrol yeasts, while yeast strains possess distinctive characteristics and have substantially different action mechanisms.

## 1. Introduction

Blueberry (*Vaccinium* spp.) is an *Ericaceae* plant [1]. Blueberry fruits are known as a superfood and show the potential to prevent chronic degenerative diseases because they contain abundant bioactive compounds with health promotion effects, including vitamin C (VC), polyphenols, micronutrients, and organic acids [2,3]. Therefore, blueberries have been cultivated on a large scale globally and are popular with consumers [4]. However, blueberries are susceptible to damage and pathogen contamination, limiting their utilization and economic value. Over 50% of postharvest fruit loss is caused by pathogenic fungi [5]. Blueberries are likely to be influenced by postharvest diseases resulting from a variety of fungal pathogens, including *Botrytis cinerea*, *Alternaria* spp., and *Penicillium* spp. [6,7]. Thus, it is of necessity to use different postharvest pathogen control approaches to lower the annual postharvest economic losses of blueberry fruit. 

Currently, postharvest disease management remains to be largely dependent on the use of artificial chemical fungicides [8]. Nevertheless, the abuse of such artificial fungicides causes chemical residues and environmental pollution and affects human health [9]. As a result, biological control agents (BCAs), the eco-friendly and safe approach, are rapidly being developed to progressively reduce artificial fungicide application [10]. Many microorganisms (fungus, bacteria, and yeast) have been investigated to control postharvest pathogenic fungi, including *Aureobasidium pullulans* S-2 [11], *Sporidiobolus pararoseus* [12], *Debaryomyces hansenii* [13], *Meyerozyma* sp. [14,15], *Bacillus subtilis* [16,17], and *Bacillus amyloliquefaciens* [18,19]. These antagonistic microorganisms can not only effectively control postharvest decay, but also enhance the postharvest fruit storage quality to some extent [20,21,22]. Additionally, probing the mechanism of action of antagonistic microorganisms using advanced techniques of microbiology, molecular biology, and new omics-based technologies is important to improve the performance while establishing criteria to screen for novel isolates [23]. Relative to additional microbial antagonists, yeasts possess numerous advantages, resulting in them being more appropriate as BCAs, including low nutritional demands, survival in unfavorable environments, high performance to resist various pathogens on diverse substances, and compatibility with commercial processing processes [24,25]. Up to now, the mechanisms underlying antagonistic yeasts like *Metschnikowia pulcherrima*, *Metschnikowia citriensis*, *Candida oleophila*, *Pseudozyma antarctica*, *Saccharomyces cerevisiae*, *Wickerhamomyces anomalus*, and *Aureobasidium pullulans* for postharvest-specific pathogenic fungi in different fruits have been demonstrated [26,27,28,29]. Some of them have been patented as BCAs [30,31]. Those potential mechanisms underlying antagonistic yeasts include competition for space and/or nutrients (like nitrogen and iron), the production of antifungal volatile organic compounds (VOCs), the release of hydrolases (glucanases, chitinases, and proteases), parasitism, biofilm generation, the involvement of oxidative stress, or host resistance development [24,32,33,34]. Nonetheless, little is known concerning biocontrol over postharvest diseases in blueberry fruits using their main pathogenic fungi. The antagonistic yeast-related postharvest disease control mechanisms in blueberry fruits are unclear.

This study isolated three strains of antagonistic yeasts from the surface of healthy blueberry fruit, which were identified as *Papiliotrema terrestris*, *Hanseniaspora uvarum*, and *Rhodotorula glutinis* through PCR amplification as well as partial sequencing of 5.8 S ribosomal DNA (rDNA) and internally transcribed spacer (ITS) regions. This study focused on (1) investigating the biocontrol effects of the three yeasts on the main pathogenic fungi (*B. cinerea* and *A. alternata*) of blueberry fruits; (2) characterizing the population dynamics for *P. terrestris, H. uvarum, and R. glutinis* within wounds on blueberry fruit preserved at 20 °C or 4 °C; (3) exploring the possible mechanisms (competition for space and/or nutrients, biofilm formation, or the extracellular lytic enzymes and VOCs generation) involved in yeast species’ biocontrol activity in resisting *B. cinerea* and *A. alternata* in vitro and in vivo; and (4) analyzing the impacts of three yeasts on the natural decay development and quality index in blueberry fruits. 

## 2. Materials and Methods

### 2.1. Fruits

Blueberry (*Vaccinium corymbosum* L.) fruits of the cultivar ‘Bluecrop’ were collected in a conventional orchard (Pinggu, Beijing, China) at the commercial maturity stage and delivered within 4 h to the laboratory. After selecting fruits in line with the consistent size and maturity, without pathological and physical damage, the selected fruits were preserved at 4 °C until analysis. 

### 2.2. Pathogen Isolation

Based on morphologies and ITS region sequencing, *Botrytis cinerea* and *Alternaria alternata* were separated from decayed blueberry fruit surfaces, identified, and shown to be highly pathogenic to blueberry fruits with Koch postulates. Afterwards, the pathogens were maintained in the potato dextrose agar medium (PDA consisting of 200 g/L boiled potato extract, 20 g/L agar, and 20 g/L glucose) at 4 °C before use. In addition, the 5 d culture surface was flooded on the PDA medium using sterile distilled water (SDW) including 0.1 g/kg Tween-80 and the filtrate at 25 °C and filtered with the four-layer sterile cheesecloth to collect pathogen spores. A hemocytometer was employed to determine spore concentrations, which were adjusted using SDW as required. 

### 2.3. Yeast Preparation

Antagonistic yeasts, *P. terrestris*, *H. uvarum*, and *R. glutinis*, were originally separated from healthy blueberry fruit surfaces. Then, those isolates were identified through morphologies, physiological characteristics, and DNA sequencing. The yeasts were maintained using nutrient yeast dextrose agar medium (NYDA: 5 g/L yeast extract, 8 g/L beef extract, 20 g/L agar, at 10 g/L dextrose) at 4 °C for further analysis. To prepare the yeast cell suspensions, these yeasts were grown in NYDB medium (agar-free NYDA, 50 mL) with shaking for 24 h (200 rpm) at 28 °C, respectively. Afterwards, the samples were centrifuged at 6000× *g* for 10 min at 4 °C to collect the cells, which were subsequently rinsed by SWD twice and adjusted using a hemocytometer to the concentrations needed in the following experiments. 

### 2.4. Biocontrol Effect of the Three Yeasts on B. cinerea and A. alternata in Blueberry Fruits

The effects of the yeasts on *B. cinerea* and *A. alternata* in blueberry fruits were assayed as described by Zhou et al. [35], with slight modifications. After selection, fruit samples were subjected to 1 min surface disinfection with 2% (*v*/*v*) sodium hypochlorite, rinsed by SDW, and air-dried before utilization. Afterwards, the tip of a sterile dissecting needle was used to make one uniform wound (depth, 3 mm; width, 3 mm) at the equator of every sample. Then, yeast suspension aliquots (10 μL, 1 × 10^8^ CFU mL^−1^) were added at each wound site, with the exception of the control group receiving sterile water treatment. After 4 h, suspensions of two pathogens (5 μL, 1 × 10^5^ spores mL^−1^) were inoculated at every wound site. Then, blueberry fruits were air-dried and preserved within polyethylene-lined plastic boxes at 20 °C. The boxes were sealed using a plastic wrap to maintain the 85–95% relative humidity (RH). Disease incidence and lesion diameter were calculated daily using the following equation: Diseased incidence (%) = (the number of diseased blueberries/the total blueberry number) × 100%, while lesion diameter (mm) was measured as average diameter within the diseased area. Each treatment was replicated 3 times with 50 fruits per replication. Meanwhile, we performed the experiment twice.

### 2.5. Antagonistic Activity on Agar Plates

The activities of yeasts on *B. cinerea* and *A. alternata* mycelial growth were assessed as described by Parafati et al. [28], with mild modifications. After orthogonal streaking of 20 μL of every yeast suspension (1 × 10^8^ CFU mL^−1^) from the Petri dish center containing the medium (pH 4.5) and PDA at a pH of 6.0, the suspensions were incubated for 48 h at 25 °C. Afterwards, the *B. cinerea* or *A. alternata* suspension (20 μL, 1 × 10^5^ spores mL^−1^) was subjected to inoculation onto the PDA plates at a distance of 2.5 cm from the yeast inoculum. Plates inoculated with the pathogen alone were utilized as the control. All plates were placed in the incubator at 25 °C for 7 d. Subsequently, growth inhibition was estimated by I% = (C − T/C) × 100, in which I% suggests inhibition against radial mycelial growth, C indicates the radial growth determined for the control, and T is the radial pathogen growth with yeast strains. Every experiment was conducted three times. 

### 2.6. Impact of Yeasts on B. cinerea and A. alternata Spore Germination In Vitro

*B. cinerea* and *A. alternata* spore germination was evaluated in potato dextrose broth (PDB) medium according to Lu et al. [17], with mild modifications. A pathogenic fungal spore suspension (1 mL, 1 × 10^7^ spores mL^−1^) was added into PDB (50 mL) containing the cell suspension of each yeast (1 mL, 1 × 10^7^ CFU mL^−1^). Meanwhile, PDA under inoculation with pathogenic fungi alone served as the control. Following 4, 6, and 8 h of inoculation on the rotary shaker (180 rpm) at 28 °C, a microscope was employed to observe around 100 spores in each replicate. The treatment was performed three times.

### 2.7. Effects of VOCs In Vitro

The VOC production ability of yeasts on *B. cinerea* and *A. alternata* was determined as described by Parafati et al. [28] and Oztekin and Karbancioglu-Guler [27], with slight modifications. Yeast suspension aliquots (20 μL, 1 × 10^8^ CFU mL^−1^) were inoculated onto plates containing PDA at a pH of 6.0 and a pH of 4.5, followed by 48 h of incubation at 25 °C. Afterwards, *B. cinerea* or *A. alternata* conidial suspension aliquots (20 μL, 1 × 10^6^ spores mL^−1^) were added in PDA prior to drying at room temperature. Subsequently, plates containing fungal conidia were covered separately with face-to-face beneath dishes including the 48 h old yeast strains. Parafilm was utilized to wrap these two plates together, with two revs around the edges to prevent air leakage, followed by a 9-day incubation period at 25 °C. Meanwhile, plates under fungal spore inoculation alone acted as the control. The fungal mycelial growth inhibition rate of yeasts was determined according to the description in Section 2.5. All experiments were carried out three times.

### 2.8. Biofilm Formation

Jin et al.’s [36] and Liu et al.’s [26] methods were used to assess biofilm formation after slight revision. After the growth of yeasts within the yeast nitrogen base (YNB) medium containing 100 mM glucose at 28 °C, cells were collected, rinsed twice using PBS (pH 7.2), and resuspended to 10^7^ CFU mL^−1^ in the YNB medium containing 100 mM glucose. Afterwards, 100 μL aliquots were subject to inoculation for 3 h into 96-well polystyrene plates at 28 °C using the shaker at 75 rpm, with three wells being prepared for each treatment. At the same time, yeast-free cells served as the control. After adhesion, PBS (150 mL) was added to wash the cells, while YNB (100 mL) was then added to every well, followed by 48 h of incubation of the plates at 28 °C at 75 rpm. The freshly prepared medium was added to replace the original medium every day. Each experiment was conducted twice.

Following 3 and 48 h of incubation, PBS was added to rinse the wells, while 0.4% aqueous crystal violet solution (100 μL) was used for staining for 45 min. After being washed with SDW four times, cells were immediately destained using 95% ethanol (200 μL). After 45 min, the destaining solution (100 μL) was added into another polystyrene 96-well plate. The solution crystal violet amount was determined at 590 nm. In addition, the absorbance values for the controls were subtracted from the values for the test well to minimize background interference.

### 2.9. Extracellular Lytic Enzymes Activities

We tested the extracellular lytic enzyme (chitinase and *β*-1,3-glucanase) production abilities of yeasts when they were cultured with pathogen cell wall preparations (CWPs) in vitro. CWPs were performed according to the description by Saligkarias et al. [37]. The *B. cinerea* and *A. alternata* mycelia were collected after incubation within 100 mL PDB at 25 °C for 7 days using 4-fold cotton gauzes. Next, the mycelia were rinsed four times using deionized water and filtered with Whatman No.1 filter paper, followed by centrifugation at 500× *g* for 2 min. The supernatant was removed, and the mycelial mat was subjected to 10 min of sonication using a probe-type sonicator and another 5 min of centrifugation at 500× *g*. After the removal of the supernatant, pellet resuspension was completed within SDW. Later, Tris/HCl buffer at the equivalent amount (50 mM and pH 7.2) was added to resuspend the crushed mycelia, followed by 15 min of centrifugation at 2000× *g* to discard the supernatant. Afterwards, the pellet was centrifuged and resuspended for 3 cycles successively, followed by freezing in liquid N_2_, lyophilization, and preservation at −20 °C before analysis. Yeast strains were cultured within modified Lilly–Barnett minimal salt medium consisting of 2 mg/mL CWP as the only source of carbon. Then, the culture medium (100 mL) within the 250 mL Erlenmeyer flask was inoculated onto the rotary shaker at 200 rpm and 25 °C for 0, 24, 48, 72, and 96 h. Every culture was centrifuged for 5 min at 8000× *g* to obtain the filtrate, while the supernatant was collected to analyze enzyme activities. 

The chitinase and *β*-1,3-glucanase activities were analyzed as described by Zhou et al. [38]. To measure the activity of chitinase, 0.05 mL of crude enzyme solution was added to 0.5 mL of a 10 mg mL^−1^ colloidal chitin suspension and 0.95 mL of 100 mmol L^−1^ sodium acetate buffer (pH 5.2). The mixture was then incubated at 37 °C for 1 h. Following the initial incubation, 0.1 mL of 3% desalted snail enzyme was added to the mixture, and incubation is was at 37 °C for an additional 70 min to facilitate the release of *N*-acetylglucosamine monomers. After incubation, the reaction mixture was promptly removed from the incubator and 0.2 mL of 0.6 mol L^−1^ potassium tetraborate was added. The mixture was then boiled in a water bath for 3 min and rapidly cooled. Once cooled, 2 mL of a diluted 4-(dimethylamino) benzaldehyde (DMAB) solution (Sigma-Aldrich, St. Louis, MO, USA) (prepared by dissolving 10 g DMAB in 87.5 mL acetic acid and 12.5 mL 10 M hydrochloric acid, stored at 4 °C, and diluted 5 times with acetic acid before use) was added. The mixture was then incubated at 37 °C for 30 min for color development, followed by the measurement of the absorbance at 585 nm. A boiled enzyme solution for 5 min was used as a control. The amount of *N*-acetylglucosamine produced was calculated based on a standard curve. The chitinase activity was denoted as a unit, with one unit indicating the enzyme activity needed to catalyze one micromole of *N*-acetylglucosamine generation/hour/milligram protein. To assess the activity of *β*-1,3-glucanase, 100 µL of enzyme solution was mixed with 100 µL of a 0.4% solution of laminarin (Sigma-Aldrich, St. Louis, MO, USA) and incubated at 37 °C for 45 min. After incubation, 1.8 mL of distilled water and 1.5 mL of DNS reagent were added to the mixture, which was then heated in a boiling water bath for 5 min to terminate the enzymatic reaction. The solution was quickly cooled post-boiling, and the colored reaction mixture was diluted to 25 mL with distilled water and mixed thoroughly, and the absorbance was measured at 540 nm. The crude enzyme solution was boiled for 5 min as a control. The amount of reducing sugars produced was calculated using a standard curve. *β*-1,3-glucanase activity was denoted as a unit, with one unit suggesting the enzyme amount needed to generate the reducing sugar equal to one micromole of glucose/hour/milligram protein. This experiment was carried out twice, with three replicates being performed. 

### 2.10. Colonization of Fruit Wounds

The sterile needle (one wound per fruit) was used to wound the superficially sanitized blueberry fruits (depth, 3 mm; width, 3 mm). Every yeast suspension (10 μL, 1 × 10^6^ CFU mL^−1^) was inoculated on the wounds, respectively. Then, the blueberry fruits were put onto the plastic packaging trays for incubation at 20 or 4 °C. Samples which contained the entire wound were obtained with a sterile cork borer at 0, 2, 4, 6, and 8 days at 20 °C or at 0, 4, 8, 12, and 16 days at 4 °C after inoculation (following 1 h of incubation as time 0). Later, a mortar was utilized to grind every sample before being pestled into PBS (10 mL, pH 7.2), followed by the spreading of serial 10-fold dilutions (50 μL) onto NYDA plates. At 48 h postincubation at 28 °C, the colony number was calculated and represented by Log10 CFU according to the fresh tissue weight. Three replicates were set for every treatment. Then, 5 fruits under each replicate. This experiment was conducted twice.

### 2.11. The Impact of Yeasts on the Natural Infection and the Quality of Blueberry Fruits 

To evaluate the impact of *P. terrestris*, *H. uvarum*, or *R. glutinis* on the natural infection and the quality of blueberry fruits, intact fruits were drenched within the yeast cell suspensions (1 × 10^8^ CFU mL^−1^) for 3 min, and later subjected to incubation within the humid incubator at 20 °C and under 85–95% RH conditions. Fruit samples dipped with SDW served as the control. On days 0, 3, 6, and 9 post-treatment, fruits were harvested. There were a total of 300 fruits utilized for every treatment, and 3 replicates were arranged randomly. The fruit decay rate was determined by (rot/total fruit number) × 100%, in which “rot” indicates the leakage of fruit juice, evident rot on the fruit surface, or obvious fungal growth. Weight loss (%) can be determined as follows: [(A – B)/A] × 100, in which A stands for the sample weight prior to treatment, whereas B indicates the final sample weight. Subsequently, we determined VC, total soluble solid (TSS), and titratable acidity (TA) contents within the fruit juice [17,39]. A total of 10.0 g fresh sample of blueberry fruits per replication was homogenized, and the homogenate was centrifuged to obtain clear juice. The TSS level was explored with the portable refractometer and is represented as a percentage. VC and TA contents were analyzed by the titration method using 0.1 M NaOH and 2, 6-dichlorophenol indophenol. The results of VC and TA contents are represented as mg/100 g FW and the percentage of citric acid equivalents, respectively.

### 2.12. Statistical Analysis

The data obtained in this study were analyzed with SPSS Software (Version 22.0, SPSS Inc., Chicago, IL, USA). ANOVA was performed for data analysis to examine differences among treatments. Duncan’s multiple range tests were adopted for mean separations. *p* < 0.05 stood for statistical significance. The data were represented using means ± standard deviations.

## 3. Results

### 3.1. Biocontrol Efficacy of Yeasts in B. cinerea and A. alternata In Vivo

**Figure 1:**
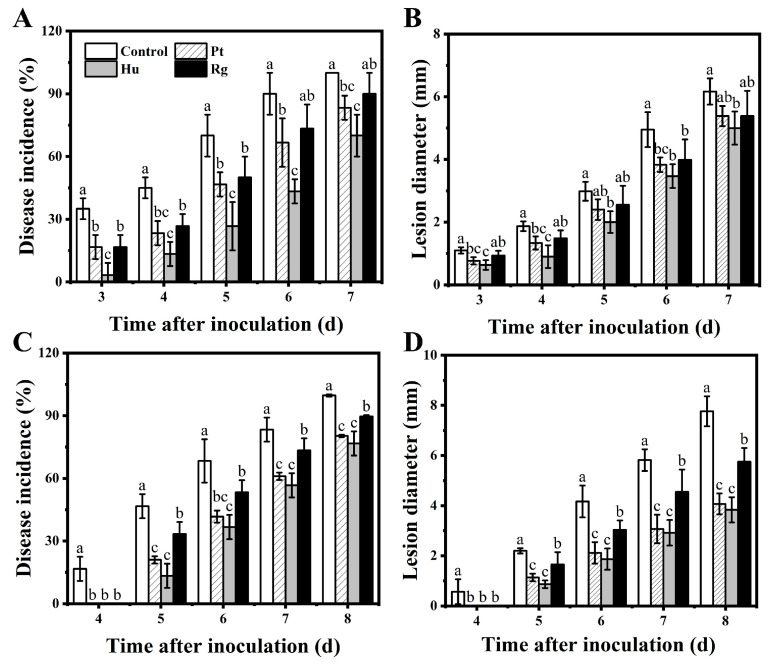
In vivo antagonistic activities of *P. terrestris*, *H. uvarum*, and *R. glutinis* in hindering *B. cinerea* and *A. alternata* on blueberry fruits. Impact of yeasts are referring to (**A**) disease incidence and (**B**) lesion diameter resulting from *B. cinerea*, and (**C**) disease incidence and (**D**) lesion diameter caused by *A. alternata* after inoculation at 20 °C. Pt, Hu, and Rg in the figure denote *P. terrestris*, *H. uvarum*, and *R. glutinis*, respectively. Data in columns with different letters at same time are of significant difference based on Duncan’s multiple range test (*p* < 0.05).

In accordance with values of lesion diameter and disease incidence of blueberry fruits, *B. cinerea* had enhanced pathogenicity on blueberry fruits relative to *A. alternata*. Compared with the control group, all yeast strains could suppress disease occurrence and development within blueberry fruits to different degrees after inoculation with pathogenic fungi (Figure 1). For retarded *B. cinerea* growth (relative to the control), the *H. uvarum* strain was more efficient than *P. terrestris* and *R. glutinis*, especially in the late storage period (Figure 1A,B). After 6 days of inoculation with *B. cinerea*, disease incidence in the fruits of the *H. uvarum*, *P. terrestris*, and *R. glutinis* groups decreased by 46.67%, 23.33%, and 16.67% compared with the control group, respectively. Similarly, the control of *A. alternata* obtained the same result. However, the three strains showed stronger inhibition against *A. alternata* in vivo (Figure 1C,D). After 8 days of inoculation with *A. alternata*, the lesion diameters of fruits in the *H. uvarum*, *P. terrestris*, and *R. glutinis* groups were 3.83, 4.06, and 5.75 mm, respectively, significantly decreased relative to the control group (7.76 mm).

### 3.2. Function of Yeasts in B. cinerea and A. alternata Mycelial Growth In Vitro

As suggested by the function of yeasts in *B. cinerea* and *A. alternata* mycelial growth, the tested yeasts could suppress pathogen mycelial growth (Table 1). *P. terrestris*, *H. uvarum*, and *R. glutinis* all exhibited higher effects on suppressing *B. cinerea* and *A. alternata* growth. Moreover, compared with pH 6.0 culture medium, all the tested yeasts showed a stronger inhibition effect against *B. cinerea* and *A. alternata* mycelial growth at pH 4.5 culture medium. Among these strains, *H. uvarum* significantly inhibited mycelial growth in both pathogens compared with *P. terrestris* and *R. glutinis* (*p* < 0.05). Although *R. glutinis* had the weakest inhibitory effect on pathogens among the three tested strains, its inhibitory rates against mycelial growth in *B. cinerea* and *A. alternata* could still reach 31.85% and 26.73%, respectively, in the medium pH 4.5 after being incubated for 7 days.

### 3.3. Function of Yeasts in B. cinerea and A. alternata Spore Germination In Vitro

**Figure 2:**
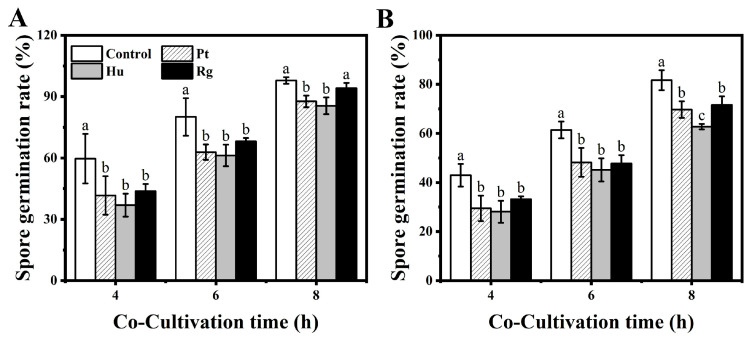
Effect of *P. terrestris*, *H. uvarum*, and *R. glutinis* on spore germination of *B. cinerea* (**A**) and *A. alternata* (**B**). Pt, Hu, and Rg in the figure denote *P. terrestris*, *H. uvarum*, and *R. glutinis*, respectively. Data in columns with different letters at same time are of significant difference in line with Duncan’s multiple range test (*p* < 0.05).

As observed from Figure 2, all of the tested strains showed stronger antagonistic effects against *B. cinerea* and *A. alternata* spore germination compared with the control, especially within 4–6 h of co-cultivation. The spore germination rates of the pathogenic fungi were not significantly different in treatment groups containing the three test strains within 4–6 h of co-cultivation, but they were all notably lower than the rate in the control group (*p* < 0.05). Following 8 h of co-cultivation, *R. glutinis* had weakened inhibition against *B. cinerea* spore germination, while the difference was not significant compared with the control. Despite this, inhibition against *A. alternata* spore germination by the three strains remained effective, with *H. uvarum* being the most effective. The spore germination rates were 85.51% and 62.73% for *B. cinerea* and *A. alternata* at 8 h following co-culture with *H. uvarum*.

### 3.4. The Antagonistic and Inhibitory Activities of VOCs In Vitro

Table 2 exhibits the inhibition rates of VOCs generated through three yeasts against pathogenic mycelial growth. Similarly, *B. cinerea* and *A. alternata* were most significantly inhibited by VOCs at a pH of 4.5. The VOCs produced by *H. uvarum* had the strongest inhibitory effect on pathogens. After 9 days of co-cultivation at a pH of 4.5, the mycelial inhibition rates of VOCs generated by *H. uvarum* against *B. cinerea* and *A. alternata* reached 42.33% and 29.26%, respectively, significantly increased relative to those other two strains (*p* < 0.05). At a pH of 6.0, the inhibition rates of VOCs generated through *H. uvarum* and *P. terrestris* against *B. cinerea* and *A. alternata* mycelial growth were not significantly different, but both were significantly stronger than that of *R. glutinis* (*p* < 0.05).

### 3.5. Biofilm-Forming Capacity

**Figure 3:**
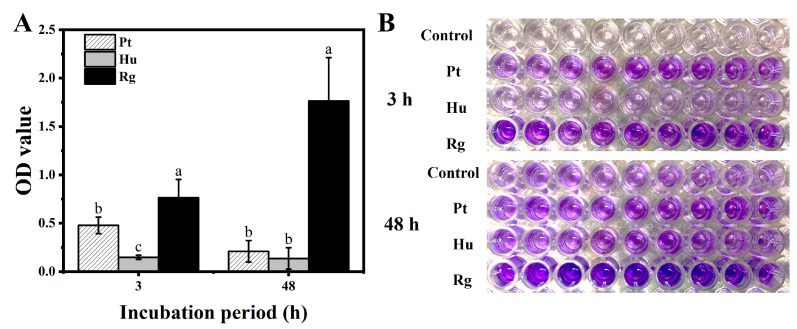
The yeasts’ biofilm formation ability. The biofilm formation was tested for *P. terrestris*, *H. uvarum*, and *R. glutinis*. Pt, Hu, and Rg in the figure denote *P. terrestris*, *H. uvarum*, and *R. glutinis*, respectively. The OD values (**A**) and the phenomenon of color development (**B**) suggest the capacity of yeast cells to adhere to the polystyrene plates. The data in the columns with different letters at the same time are of significant difference based on Duncan’s multiple range test (*p* < 0.05).

All of the tested yeast strains were more or less capable of forming biofilms under the culture condition assayed (Figure 3). *P. terrestris* adhered to the polystyrene plates regardless of repeated washes, exhibiting an OD value of 0.48 after 3 h of incubation and a value of 0.21 after 48 h of incubation. *H. uvarum* showed a decreased biofilm generation capacity, because it almost did not adhere to the polystyrene plates (OD < 0.2). Compared with the other two strains, *R. glutinis* had the strongest biofilm formation ability (*p* < 0.05). The OD value of *R. glutinis* reached 0.76 after 3 h of incubation and rapidly increased to 1.76 after 48 h of incubation.

### 3.6. Extracellular Lytic Enzyme Activity

**Figure 4:**
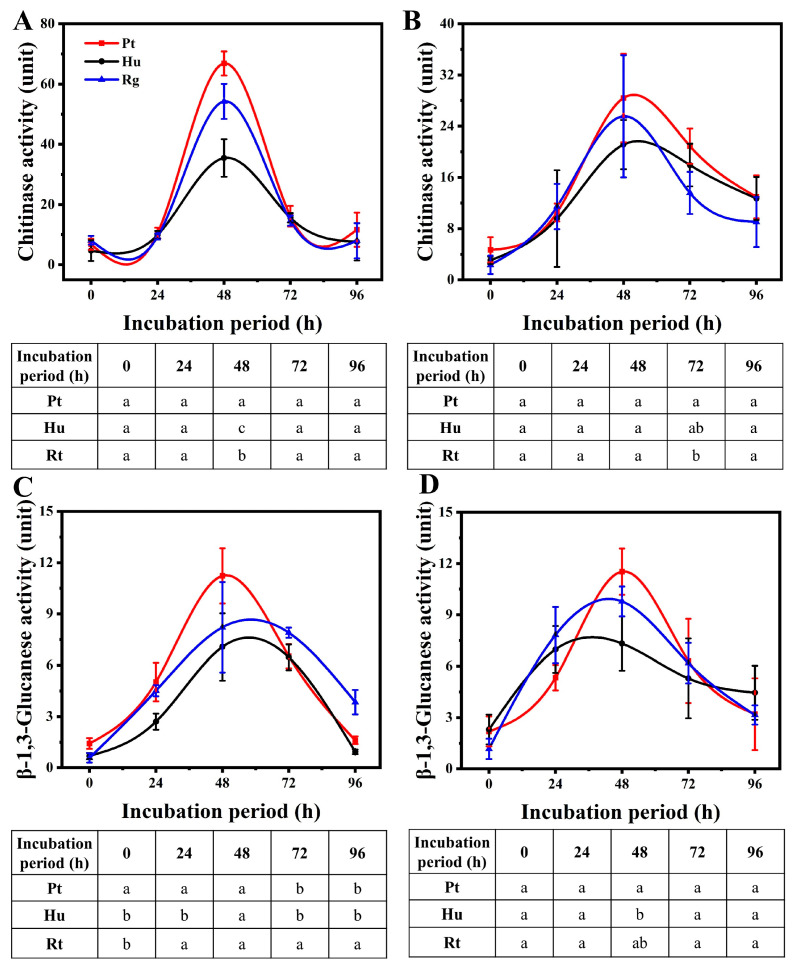
Extracellular lytic enzyme activity of yeasts. Chitinase activities of yeasts in presence of cell wall preparations (CWPs) of *B. cinerea* (**A**) and *A. alternata* (**B**). *β*-1,3-glucanase activities of yeasts in presence of CWPs of *B. cinerea* (**C**) and *A. alternata* (**D**). Pt, Hu, and Rg in figure denote *P. terrestris*, *H. uvarum*, and *R. glutinis*, respectively. Different letters at same time indicate statistically significant differences based on Duncan’s multiple range test (*p* < 0.05).

The three tested yeasts generated extracellular chitinase and *β*-1,3 glucanase at once following induction if the yeasts were grown with CWPs of *B. cinerea* or *A. alternata* being the only carbon source, and the activities of both enzymes peaked after 48 h of induced incubation, followed by a gradual decrease (Figure 4). Comparatively, *P. terrestris* was the strongest producer of extracellular hydrolases, *R. glutinis* was the second strongest, and *H. uvarum* was the weakest. Relative to *A. alternata*, *P. terrestris, H. uvarum*, and *R. glutinis* had stronger chitinase activity under the induction of *B. cinerea* after incubation for 48 h, with chitinase activities of 66.84, 35.43, and 54.32 U, respectively. Meanwhile, the activities of chitinase produced by the three tested yeasts under *A. alternata* induction were only 29.41, 21.12, and 25.56 U, respectively (Figure 4A,B). However, the *β*-1,3 glucanase activities generated in the three strains were not influenced by *B. cinerea* and *A. alternata*; in addition, they had similar activities within both modified mediums (with CWPs of *B. cinerea* or *A. alternata* being the only carbon source) (Figure 4C,D).

### 3.7. Wound Site Colonization

**Figure 5:**
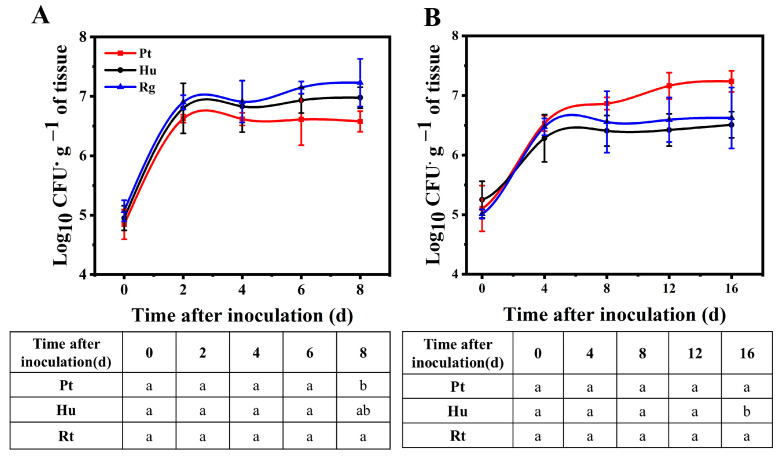
Population dynamics of *P. terrestris*, *H. uvarum*, and *R. glutinis* in wounds of blueberry fruits at 20 °C (**A**) and 4 °C (**B**). Hu, Pt, and Rg in figure denote *P. terrestris*, *H. uvarum*, and *R. glutinis*, respectively. Different letters at same time indicate statistically significant differences based on Duncan’s multiple range test (*p* < 0.05).

Figure 5 displays the population dynamics for *P. terrestris*, *H. uvarum*, and *R. glutinis* within fruit wounds. At 20 °C, the yeast population showed rapid growth, particularly 2 days after inoculation. The population of *P. terrestris* at the wound reached a maximum (6.62 lg CFU g^−1^ of tissue) after 2 days of inoculation and later remained stable. However, *H. uvarum* and *R. glutinis* still slowly increased their populations after 2 days of rapid growth and reached a maximum (6.97 and 7.23 lg CFU g^−1^ of tissue) after storage (Figure 5A). At 4 °C, *P. terrestris* revealed a stronger ability to colonize fruit wounds. In particular, after the initial 4 days of rapid growth, the *P. terrestris* population still increased in number. Correspondingly, the population numbers of *H. uvarum* and *R. glutinis* grew rapidly at the beginning and then were at a relatively stable level. At 16 days after inoculation, the population number of *P. terrestris* was 7.24 lg CFU g^−1^ of tissue, which is significantly increased relative to *H. uvarum* and *R. glutinis* (6.51 and 6.62 lg CFU g^−1^ of tissue; *p* < 0.05) (Figure 5B).

### 3.8. Effects of H. uvarum, P. terrestris, and R. glutinis on Natural Disease Incidence and Quality of Blueberry Fruits 

The effects of *H. uvarum*, *P. terrestris*, and *R. glutinis* treatment on the natural infection and the quality of the fruits during storage at room temperature are shown in Table 3 and Figure S1. Yeast-soaking treatments obviously decreased the fruits’ natural disease incidence and weight loss (*p* < 0.05). *H. uvarum* exerted the best control effect on fruit decay, followed by *P. terrestris* and *R. glutinis*. After storage for 9 days, the fruit natural incidence in the *H. uvarum* treatment group was only 21.67%, significantly decreased relative to the control group (60.00%) and the *P. terrestris-* and *R. glutinis*-treated groups (33.33% and 39.67%) (*p* < 0.05). Regarding the control of fruit weight loss, *H. uvarum* and *R. glutinis* were equally effective (*p* > 0.05) and both were superior to *P. terrestris* (*p* < 0.05). Meanwhile, the three yeast treatments were able to increase the TSS and VC contents and decrease the TA content of fruits to different degrees. This indicates that yeast treatments improve the flavor and nutritional quality of the fruits.

## 4. Discussion

Microbial antagonists can be generally separated in nature; in addition, yeasts localized on the surface of living plants can generate communities with the highest population number and greatest diversity due to the abundant nutrient sources generated in living plants [26,28]. In the present study, we screened three yeast strains (*P. terrestris*, *R. glutinis*, and *H. uvarum*) from the surface of healthy blueberry fruits owing to their different phenotypic characteristics, which have been reported to show excellent biocontrol efficacy against certain postharvest pathogenic fungi in vivo or in vitro in previous studies [33,40,41]. The data from this study indicated that the application of *P. terrestris*, *R. glutinis*, and *H. uvarum* significantly decreased postharvest disease incidence and lesion diameter in blueberry fruits resulting from *B.cinerea* and *A.alternata* (Figure 1). Furthermore, the natural disease incidence in blueberry fruits was significantly decreased by the three yeasts after treatment at 20 °C for 9 days. In the meantime, the quality indexes of the fruits were improved by these three yeast strains (Table 3). These results imply that these three yeasts can be developed into commercial preservatives. 

To further comprehend the action mechanisms of these yeasts as BCAs for blueberry postharvest diseases, this study explored their diverse mechanisms in vivo and in vitro. Competition for space/nutrient is identified as the main action mechanism of yeast antagonistic microorganisms in resisting postharvest fungal pathogens [34]. Yeast is capable of competing for restricted nutrition against pathogens in vitro or in the wound site and can inhibit pathogen growth, while it usually leaves the pathogen alive [42]. In this study, three strains of yeast were found to inhibit spore germination as well as mycelial growth in *B. cinerea* and *A. alternata*. Among them, *H. uvarum* had the strongest direct inhibitory effect on both pathogenic fungi (Figure 2, Table 1). Similar findings were reported in the study by Öztekin and Karbancioglu-Guler [43]: the inhibition rate of *H. uvarum* against the spore germination and mycelial growth of *Penicillium digitatum* could reach over 80%. In addition, some yeasts have the capability to produce antifungal VOCs. The VOCs are capable of long-distance diffusion in air and exert biological activities [44]. Relative to conventional liquid fungicides, microbial volatiles suppress pathogen growth while controlling postharvest diseases with no immediate contact [45]. The antifungal VOCs emitted by yeasts mainly include alcohols (ethanol, 2-methyl-propanol, 3-methyl-butanol, and 2-methyl-butanol), organic acids (3-methyl butanoic acid), esters (2-methylpropyl hexanoate, 3-methylbutyl hexanoate, and 3-methylbutyl pentanoate), benzene derivatives (2-phenylethanol and 2-phenyl ethyl acetate), aldehydes (trans-cinnamaldehyde), and hydrazines (1,1-dimethylhydrazine) [46]. The main mechanism underlying the antifungal effects of VOCs is the disruption of the cell wall and membrane structures, leading to intracellular lysate leakage and oxidative stress induction [47]. In this study, *H. uvarum* showed a stronger ability to secrete antifungal VOCs against *B. cinerea* and *A. alternata* compared to *P. terrestris* and *R. glutinis* (Table 2). For the antifungal VOCs generated by this yeast, the main component was ethyl acetate [40]. Based on this excellent property of *H. uvarum*, some researchers have utilized *H. uvarum* to prolong strawberry storage duration and enhance their storage quality by using VOCs [48,49]. Based on the obtained findings, the mechanisms by which *H. uvarum* controls postharvest disease in blueberry fruits are mainly competition for space/nutrient and antifungal VOC generation.

In addition to secreting antifungal VOCs, the three species of yeast were also detected to secrete exocellular lytic enzymes like chitinases and *β*-1, 3-glucanase. The two enzymes are associated with the mycelial degradation of fungi [26,27,38]. They can result in cell deformities, alterations in membrane permeability, and cytoplasmic leakage, and therefore have a vital impact on biocontrol over pathogens [50,51,52]. According to our findings, *P. terrestris* had stronger exocellular lytic enzyme activity than *H. uvarum* and *R. glutinis* when induced by the CWPs of *B. cinerea* or *A. alternata*, especially following 48 h of induced incubation (Figure 4). Biofilm formation accounts for an important part of yeast biology, which facilitates the biocontrol effect. Yeast cells contained in the complicated extracellular matrix allow for their adherence onto host surfaces more strongly [53]. Yeast cells extracted following biofilm formation can effectively inhibit pathogen growth and colonize inner wound surfaces [34,54]. In this study, *R. glutinis* was more efficient in biofilm formation in vitro compared to *H. uvarum* and *P. terrestri* (Figure 3). Moreover, *R. glutinis* maintained the highest population density in wounds in blueberry fruits at 20 °C (Figure 5A). The above findings indicate that the secretion of exocellular lytic enzymes and biofilm formation are the main mechanisms by which *P. terrestri* and *R. glutinis* control postharvest disease in blueberry fruits, respectively. 

## 5. Conclusions

To conclude, this study demonstrates that *P. terrestri*, *H. uvarum*, and *R. glutinis*, separated from healthy blueberry fruit surfaces, can be BCAs to resist *B. cinerea* and *A. alternata* infection in postharvest blueberry fruits. The mechanisms of these various yeasts are diverse, while each yeast strain possesses distinct characteristics and exhibits unique dominant mechanisms. According to our findings, three isolated biocontrol yeasts show diverse dominant mechanisms. The secretion of exocellular lytic enzymes is identified to be the main biocontrol mechanism of *P. terrestris* yeasts. Producing antifungal VOCs and competing for nutrients and space are suggested as the major biocontrol mechanisms of *H. uvarum*. Biofilm formation has a vital effect on the biocontrol efficacy of *R. glutinis*. 

## Figures and Tables

**Table 1:** In vitro antagonistic activity of yeast strains referring to mycelial growth inhibition of *B. cinerea* and *A. alternata.* Different letters in each column are of significant difference based on Duncan’s multiple range test (*p* < 0.05). Each value is mean ± standard deviation of three replicates.

Isolates	Inhibition of Mycelial Growth of *B. cinerea* (%)		Inhibition of Mycelial Growth of *A. alternata* (%)	
	pH 4.5	pH 6.0	pH 4.5	pH 6.0
*P. terrestris*	45.79 ± 2.16 b	37.01 ± 2.03 b	38.29 ± 2.41 b	30.14 ± 1.16 b
*H. uvarum*	55.25 ± 1.72 a	42.33 ± 1.44 a	43.71 ± 1.07 a	35.43 ± 1.32 a
*R. glutinis*	31.85 ± 1.70 c	26.67 ± 2.24 c	26.73 ± 1.42 c	17.64 ± 3.03 c

**Table 2:** In vitro antagonistic activity of volatile organic compounds (VOCs) produced by yeast strains toward *B. cinerea* and *A. alternata*. Different letters in each column are of statistical significance based on Duncan’s multiple range test (*p* < 0.05). Each value suggests mean ± standard deviation of three replicates.

Isolates	Inhibition of Mycelial Growth of *B. cinerea* (%)		Inhibition of Mycelial Growth of *A. alternata* (%)	
	pH 4.5	pH 6.0	pH 4.5	pH 6.0
*P. terrestris*	36.01 ± 2.03 b	28.66 ± 2.57 a	24.36 ± 1.67 b	19.65 ± 0.69 a
*H. uvarum*	42.33 ± 1.44 a	30.75 ± 1.28 a	29.26 ± 1.93 a	20.21 ± 3.42 a
*R. glutinis*	21.67 ± 2.07 c	20.85 ± 2.62 b	18.54 ± 3.03 c	12.64 ± 2.76 b

**Table 3:** Effects of yeast strains on natural infection and quality of blueberry fruits during storage at room temperature. Different letters in each column on same day are of statistical difference based on Duncan’s multiple range test (*p* < 0.05). Each value indicates mean ± standard deviation of three replicates.

Storage Time (d)	Treatment	Decay Incidence (%)	Weight Loss (%)	Total Soluble Solid (%)	Titratable Acidity (%)	Vitamin C (mg per 100 g FW)
0	Control	0.00 ± 0.00 a	0.00 ± 0.00 a	8.43 ± 0.45 a	0.40 ± 0.70 a	95.40 ± 0.26 a
	*P. terrestris*	0.00 ± 0.00 a	0.00 ± 0.00 a	8.77 ± 0.21 a	0.40 ± 0.06 a	95.66 ± 0.67 a
	*H. uvarum*	0.00 ± 0.00 a	0.00 ± 0.00 a	8.60 ± 0.15 a	0.39 ± 0.06 a	94.93 ± 0.66 a
	*R. glutinis*	0.00 ± 0.00 a	0.00 ± 0.00 a	8.63 ± 0.06 a	0.41 ± 0.03 a	95.27 ± 0.21 a
3	Control	16.67 ± 1.53 a	13.86 ± 1.41 a	9.87 ± 0.25 b	0.53 ± 0.70 a	88.30 ± 2.01 b
	*P. terrestris*	7.67 ± 2.52 b	7.66 ± 0.95 b	10.97 ± 0.70 a	0.48 ± 0.03 a	93.77 ± 0.71 a
	*H. uvarum*	6.10 ± 1.55 b	3.50 ± 0.31 c	10.6 ± 0.20 ab	0.26 ± 0.02 b	95.07 ± 0.32 a
	*R. glutinis*	8.10 ± 0.72 b	2.79 ± 0.16 c	11.1 ± 0.26 a	0.31 ± 0.05 b	93.57 ± 0.45 a
6	Control	38.33 ± 2.08 a	17.47 ± 2.14 a	7.83 ± 0.21 d	0.35 ± 0.02 a	86.57 ± 1.71 c
	*P. terrestris*	20.33 ± 2.52 b	9.98 ± 2.86 b	9.07 ± 0.11 c	0.41 ± 0.04 ab	91.83 ± 1.56 b
	*H. uvarum*	13.33 ± 5.16 c	5.31 ± 2.38 c	9.53 ± 0.31 b	0.24 ± 0.02 c	94.37 ± 0.61 a
	*R. glutinis*	23.33 ± 2.08 b	4.30 ± 0.30 c	10.2 ± 0.10 a	0.28 ± 0.06 bc	93.02 ± 0.66 a
9	Control	60.00 ± 1.00 a	24.03 ± 3.19 a	7.90 ± 0.36 c	0.26 ± 0.01 a	64.00 ± 2.40 c
	*P. terrestris*	33.33 ± 5.77 c	18.55 ± 1.03 b	9.03 ± 0.15 b	0.21 ± 0.02 b	77.73 ± 0.95 b
	*H. uvarum*	21.67 ± 2.58 d	14.31 ± 2.38 c	9.53 ± 0.15 a	0.15 ± 0.01 c	88.89 ± 2.16 a
	*R. glutinis*	39.67 ± 0.58 b	13.03 ± 0.57 c	8.23 ± 0.21 c	0.15 ± 0.02 c	78.56 ± 0.61 b

## Data Availability

The original contributions presented in the study are included in the article/Supplementary Material, further inquiries can be directed to the corresponding author.

## Supplementary Materials

The following supporting information can be downloaded at: https://www.mdpi.com/article/10.3390/foods13091334/s1, Figure S1: Pictures of representative blueberry samples were recorded during the storage period (0 and 9 days) at room temperature. Pt, Hu, and Rg in the figure denote *P. terrestris*, *H. uvarum*, and *R. glutinis*, respectively.
