# Supplementary material for: Inhibitory Effect and Potential Antagonistic Mechanism of Isolated Epiphytic Yeasts against Botrytis cinerea and Alternaria alternata in Postharvest Blueberry Fruits
Source: Foods. 2024 Apr 26;13(9):1334. doi: 10.3390/foods13091334 (PMC11083711; doi:10.3390/foods13091334)
Supplement: Supplementary file 1 [file foods-13-01334-s001.zip › foods-2960465-supplementary.pdf]

Supplementary Material

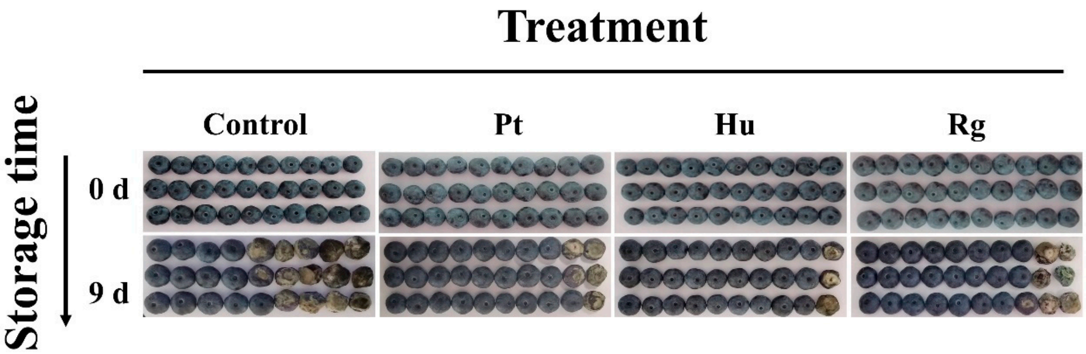

Figure S1. Pictures of representative blueberry samples were recorded during the storage period (0 and 9 days) at room temperature. Pt, Hu, and Rg in the figure denote *P. terrestris*, *H. uvarum*, and *R. glutinis*, respectively.
